# Functionalized 3D-Printed ST2/Gelatin Methacryloyl/Polcaprolactone Scaffolds for Enhancing Bone Regeneration with Vascularization

**DOI:** 10.3390/ijms23158347

**Published:** 2022-07-28

**Authors:** Guangliang Liu, Jie Chen, Xiaofang Wang, Yujiao Liu, Yufei Ma, Xiaolin Tu

**Affiliations:** Laboratory of Skeletal Development and Regeneration, Institute of Life Sciences, Chongqing Medical University, Chongqing 400016, China; cytokine01@163.com (G.L.); jiechen@stu.cqmu.edu.cn (J.C.); 2019111156@stu.cqmu.edu.cn (X.W.); yujiaoliu619@163.com (Y.L.)

**Keywords:** 3D-printing, Wnt3a, scaffold, bone regeneration, vascularization

## Abstract

Growth factors were often used to improve the bioactivity of biomaterials in order to fabricate biofunctionalized bone grafts for bone defect repair. However, supraphysiological concentrations of growth factors for improving bioactivity could lead to serious side effects, such as ectopic bone formation, radiculitis, swelling of soft tissue in the neck, etc. Therefore, safely and effectively applying growth factors in bone repair biomaterials comes to be an urgent problem that needs to be addressed. In this study, an appropriate concentration (50 ng/mL) of Wnt3a was used to pretreat the 3D-bioprinting gelatin methacryloyl(GelMA)/polycaprolactone(PCL) scaffold loaded with bone marrow stromal cell line ST2 for 24 h. This pretreatment promoted the cell proliferation, osteogenic differentiation, and mineralization of ST2 in the scaffold in vitro, and enhanced angiogenesis and osteogenesis after being implanted in critical-sized mouse calvarial defects. On the contrary, the inhibition of Wnt/β-catenin signaling in ST2 cells reduced the bone repair effect of this scaffold. These results suggested that ST2/GelMA/PCL scaffolds pretreated with an appropriate concentration of Wnt3a in culture medium could effectively enhance the osteogenic and angiogenic activity of bone repair biomaterials both in vitro and in vivo. Moreover, it would avoid the side effects caused by the supraphysiological concentrations of growth factors. This functionalized scaffold with osteogenic and angiogenic activity might be used as an outstanding bone substitute for bone regeneration and repair.

## 1. Introduction

There are more than 4 million patients with bone defects caused by severe trauma, cancer, and infection every year in the world [1]. Especially, bone defects exceeding the critical size cannot be repaired or regenerated naturally, and therefore, transplantation of bone or bone repair graft is required. Both autologous and allogeneic transplantation is limited by resource shortage and donor site complications, while allogeneic and xenotransplantation also have issues of cost and risk of disease transmission [2]. These problems have prompted the development of new bone repair biomaterials for bone defect repair. However, the insufficient bioactivity of implanted biomaterials is prone to cause repair failure and require revision of these implants [3], resulting in higher costs and shorter survival [4]. Therefore, improving the bioactivity of biomaterials emerged as a key problem that needs to be solved urgently in bone tissue engineering.

In recent years, bone repair scaffolds fabricated by implant materials such as metal (stainless steel, titanium, alloy, etc.), ceramic, or polymer are functionalized and modified by loading inorganic salts, growth factors, or stem cells to create an osteogenic environment to improve bioactivity [5]. Bioactive inorganic salts were used to simulate the osteogenic differentiation environment of stem cells, such as borate [6], bioglass [7], and calcium silicate [8], and combined with 3D-bioprinting loaded stem cells or vascular endothelial cells to construct controllable shape and structure to simulate the internal structure of bone [9,10]. Moreover, two growth factors of bone morphogenetic proteins (BMPs), BMP-2 and BMP-7, have been approved by the FDA for clinical use for more than 20 years. Grafts containing an absorbable collagen sponge and BMP-2 or BMP-7 are referred to as the Infuse device and the OP-1 device, respectively. There is little debate about their osteogenic potential [11]. However, clinical success is limited by their low stability, short half-life, and rapid diffusion from the delivery site. Supraphysiological concentration of the growth factor is often required to demonstrate efficacy [12,13]. It was reported that BMP-2 exhibits a half-life time of ~7 min in the bloodstream [14]. Effective concentrations are typically in the mg/mL (∼10^−5^ M) range, which is much more than native BMP-2 concentrations of 18.8–22 pg/mL (7.2 × 10^−13^ to 8.5 × 10^−13^ M) [12,15]. This can cause serious adverse side effects, such as ectopic bone formation, radiculitis, cervical and soft tissue swelling, etc. [12]. Consequently, BMP-7 was withdrawn from the market, and restrictions were imposed on the clinical use of BMP-2, generating the need for the development of a more efficacious treatment strategy [16].

Bone marrow stromal cells (BMSCs) are often used as seed cells in bone tissue engineering [17]. The Wnt/β-catenin signaling pathway is a key regulator of bone homeostasis. Activation of the canonical Wnt/β-catenin signaling pathway leads to osteogenic differentiation, while β-catenin inactivation leads to adipogenic or chondrogenic differentiation [18,19]. Osteogenic gene expression and mineral deposition of bone marrow stromal cell line ST2 grown on the surface of titanium/titanium alloy were dramatically increased by activating the Wnt signaling with a chimeric peptide [20]. Studies based on the synergistic induction of angiogenesis and bone formation by multiple cell cultures have been practiced for many years. Vascularization provides blood supply to the bone defect, which carries nutrients and oxygen to improve the bioactivity of the bone repair scaffold, resulting in vascularized functional bone formation [21,22]. In the co-culture system of BMSCs and human umbilical vein endothelial cells (HUVECs) on 3D bone scaffolds, HUVECs induced osteogenic differentiation of BMSCs by activating its Wnt/β-catenin signaling [23]. However, as the number of components in a scaffold system increases, so does the complexity of construction and uncertainty in treatment outcomes [24].

It was reported that the efficacy of autografts can be traced back to BMSCs residing within the bone graft. Blocking the endogenous Wnt signaling using Dkk1 abrogates autograft efficacy whereas a brief incubation in Wnt3a reliably improves autologous bone grafting efficacy [25]. It suggests that functional modification of the implant material by activating the canonical Wnt/β-catenin signaling pathway of bone marrow stromal cells by Wnt3a in vitro can improve its bioactivity and increase the efficacy of bone regeneration while avoiding the use of supraphysiological concentrations in vivo and the side effects caused thereby. Moreover, after incubation with Wnt3a for 24 h, macrophages induced in vitro tubular pattern structures in endothelial cells resembling capillary-like vasculature via releasing angiogenic growth factor [26]. Intranasally delivered Wnt3a improves vascularized recovery after traumatic brain injury by up-regulation of the expression of VEGF [27]. Wnt3a is involved in the mechanical loading on the improvement of bone formation and angiogenesis [28]. These results provide strong evidence that Wnt3a promotes bone formation and vascularization.

**Figure 1:**
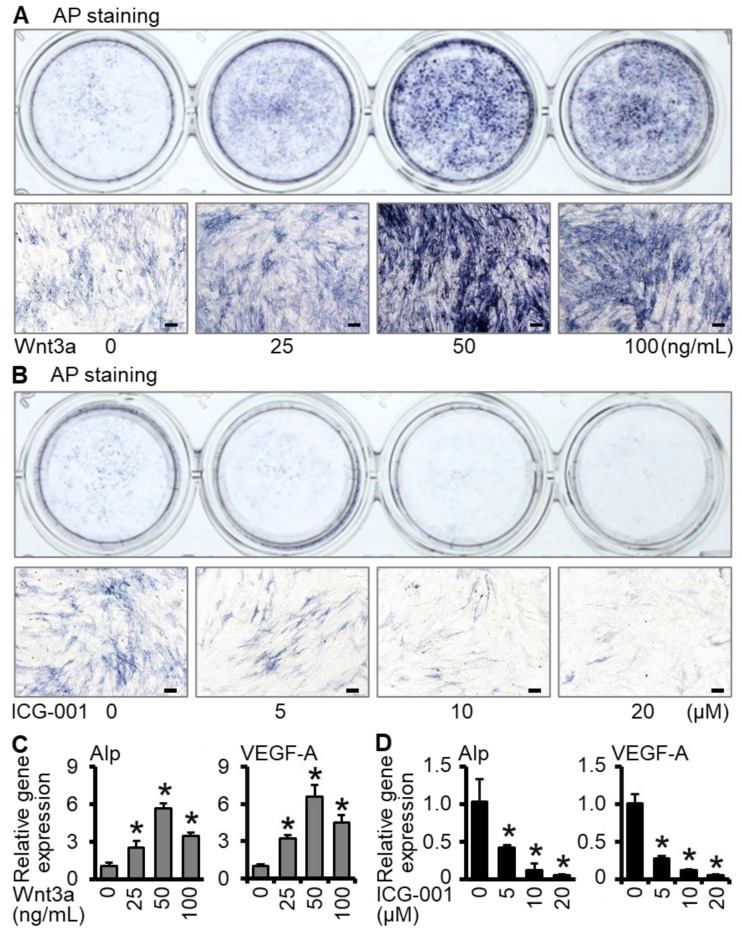
Screening of appropriate concentration of Wnt3a to promote osteogenic differentiation of ST2 cells. Under two-dimensional conditions, ST2 cells were induced by Wnt3a or ICG-001 (Wnt/β-catenin signaling inhibitor) for 24 h. AP staining and quantitative real-time polymerase chain reaction (qRT-PCR) detection were performed on the seventh day. (**A**,**B**) Alkaline phosphatase staining. (**C**,**D**) qRT-PCR detection of the expression of osteogenic differentiation gene Alp, angiogenesis factor VEGF-A. * *p* < 0.05, compared with Wnt3a 0 ng/mL or ICG-001 0 μM group by one-way ANOVA (n = 3). (Scale bars: A, 100 µm, B, 100 µm).

As autografts are limited by resource shortage, our work aims to utilize the properties of Wnt3a and autografts bone to construct an osteogenic and angiogenic bifunctional scaffold, which could achieve stable batch production by 3D printing to promote bone regeneration. In our previous study, we found that Wnt3a can promote osteogenic differentiation of bone marrow stromal cell line ST2 and up-regulation of the expression of VEGF-A, while inhibition of Wnt signaling decreased the expression of osteogenic differentiation marker and VEGF-A (Figure 1). Therefore, by combining gelatin methacryloyl (GelMA) and polycaprolactone (PCL), we envisaged the batch fabrication of ST2/GelMA/PCL scaffolds by 3D bioprinting to promote bone repair after Wnt3a pretreatment in vitro. To verify the potential of these ST2/GelMA/PCL scaffolds as bone tissue engineering biomaterials, the ability of cell survival, proliferation, osteogenic differentiation, and mineralization in ST2/GelMA/PCL scaffolds were detected. Then, the scaffolds were implanted into mice parietal critical-size bone defects to detect bone regeneration and vascular formation. These specific cellular responses are key steps in the development of functional 3D-printed bone scaffolds to promote vascularized functional bone regeneration.

## 2. Results

### 2.1. Wnt3a Promotes Osteogenic Differentiation and VEGF-A Expression of ST2 Cells in Two-Dimensional Culture

Alkaline phosphatase is an important marker of osteoblast differentiation and can be measured by alkaline phosphatase staining and quantitative real-time polymerase chain reaction (qRT-PCR) [29]. According to the results of different concentrations, the alkaline phosphatase staining in the Wnt3a group had the darkest positive staining at the concentration of 50 ng/mL (Figure 1A), and the ICG-001 group had the lightest staining at 20 μM (Figure 1B). The expression of Alp and angiogenic factor VEGF-A was consistent with the trend of alkaline phosphatase staining (Figure 1C,D). Therefore, in the subsequent experiments, the concentration of Wnt3a was selected as 50 ng/mL, and the concentration of ICG-001 was selected as 20 μM. The above results suggest that activation of Wnt signaling promotes osteogenic differentiation and vascular marker expression in ST2 cells, whereas inhibition of Wnt signaling reduces this function.

### 2.2. Preparation of ST2/GelMA/PCL Scaffolds by 3D Bioprinting and Their Biocompatibility Testing

**Figure 2:**
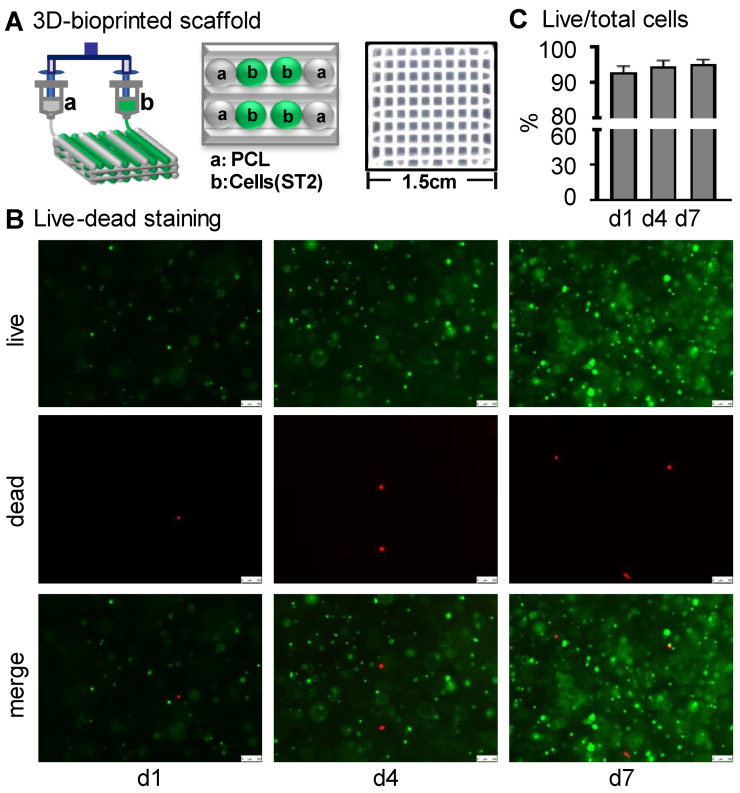
Preparation of ST2/GelMA/PCL 3D scaffold and its cell viability assay. (**A**) Schematic diagram of the 3D printing scaffold and gross pictures of the scaffold. (**B**) Fluorescence images of live/dead staining and (**C**) quantification of cell viability (the percentage between the number of living cells and total cells) in the Wnt3a pretreated scaffold on days 1, 4, and 7 (n = 3). Cell viability on days 1, 4, and 7 was not statistically different by one-way ANOVA. (Scale bar: B, 100 µm). GelMA, gelatin methacryloyl; PCL, polycaprolactone.

**Figure 3:**
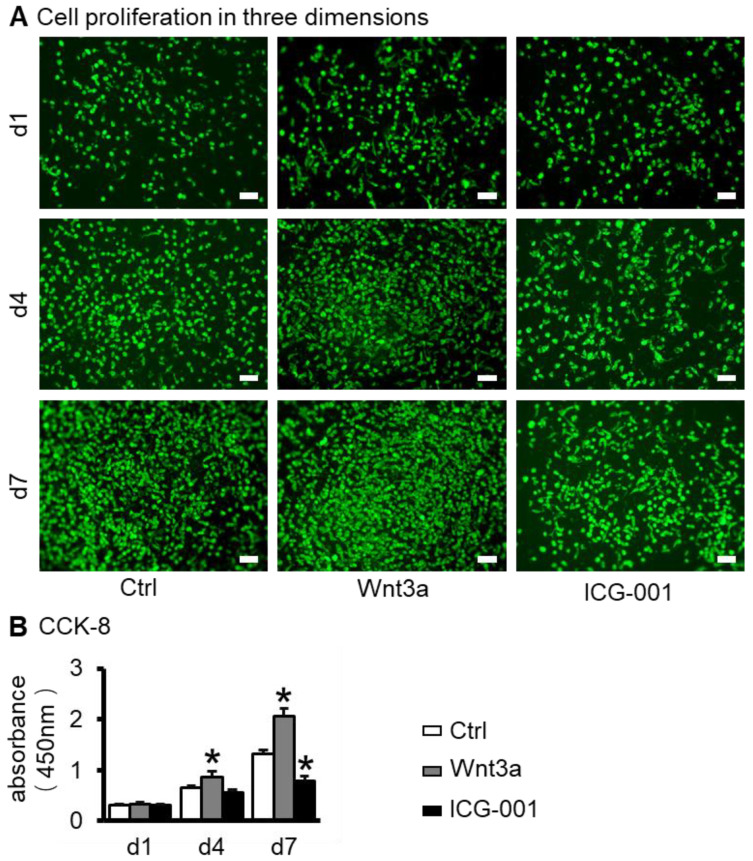
Cell proliferation activity in ST2/GelMA/PCL scaffolds. (**A**) Fluorescence photograph of the 3D scaffold. ST2 cells are labeled with Dio dye (green). (**B**) The cell proliferation detection activity in the scaffold was detected by the CCK-8 kit (n = 5). Compared with the ctrl group, * *p* < 0.05 by one-way ANOVA. (Scale bar: A, 50 µm).

The biocompatibility of bone repair scaffolds is the basis for their biological functions and is usually characterized by cell survival and proliferation [30]. Three-dimensional bone repair scaffolds of ST2/GelMA/PCL were prepared by 3D bioprinting (Figure 2A). At 1, 4, and 7 days after scaffold printing, live cells were above 92% of total cells in Wnt3a pretreated scaffolds (Figure 2B,C). The proliferation number of cells in the 3D-printed functional modules increased with time, and the proliferation activity of cells in the control group increased by 4.12 times in 7 days. Among the three groups, the Wnt3a group had the greatest proliferative activity. That was 1.57 times and 2.62 times more than the control group and ICG-001 group, respectively, on the seventh day (Figure 3A,B).

### 2.3. Wnt3a Pretreating ST2/GelMA/PCL Bone Repair Scaffold Enhanced Osteogenic Differentiation

**Figure 4:**
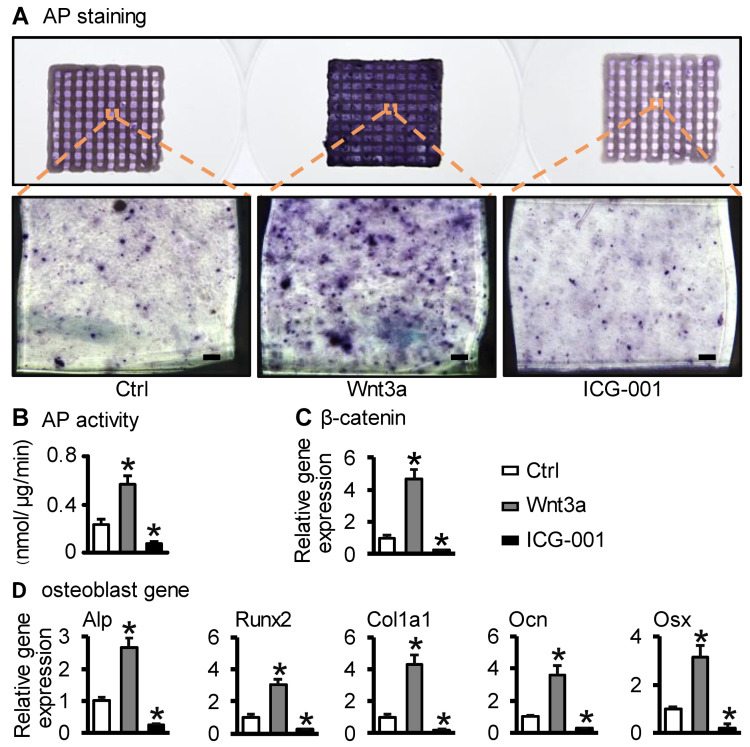
Wnt3a pretreatment enhanced osteogenic differentiation of ST2/GelMA/PCL scaffolds. The test was performed on the seventh day of scaffold culture. (**A**) Alkaline phosphatase staining and (**B**) Alkaline phosphatase activity quantification. (**C**) The expression of the key factor β-catenin of Wnt signaling and (**D**) osteogenic marker genes (Alp, Runx2, Col1a1, Ocn, Osx). Compared with the ctrl group, * *p* < 0.05 by one-way ANOVA. n = 3. (Scale bar: A, 100 µm).

Alkaline phosphate staining, quantification of alkaline phosphate biochemical activity, and osteogenic marker mRNA were used to detect the level of osteogenic differentiation. On the seventh day of scaffold culture, compared with the control group, the positive staining of alkaline phosphatase in the Wnt3a group was the deepest, followed by the control group, and the weakest in the ICG-001 group (Figure 4A). Compared with the control group, the alkaline phosphatase activity of the Wnt3a group (Wnt3a group vs. ctrl group: (0.57 ± 0.07) nmol/μg/min vs. (0.23 ± 0.04) nmol/μg/min, *p* < 0.05) was significantly increased, while it was significantly decreased in the ICG-001 group ((0.08 ± 0.02) nmol/μg/min, *p* < 0.05) (Figure 4B). qRT-PCR detection showed that the expression of β-catenin (the key factor of Wnt signaling) and osteogenic differentiation genes (Alp, Osx, Runx2, Col1a1, and Ocn) were up-regulated in the Wnt3a group, but decreased in the ICG-001 group, compared with the control group (Figure 4C,D). These suggest that activating Wnt signaling enhanced the osteogenic differentiation of ST2 cells in the scaffold while inhibiting Wnt signaling decreased the osteogenic differentiation.

### 2.4. Wnt3a Pretreating Enhances the Mineralization of ST2 Cells in Bone Repair Scaffolds

**Figure 5:**
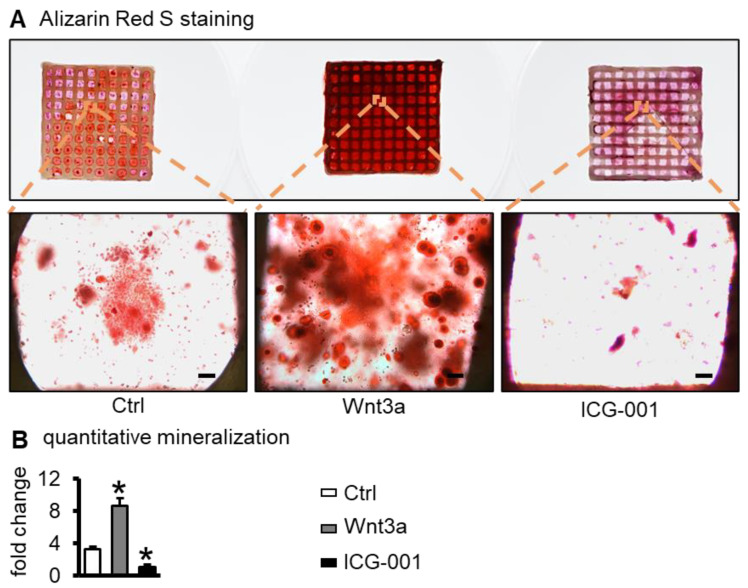
Wnt3a pretreatment enhanced mineralization of ST2/GelMA/PCL scaffolds. After the seventh day of scaffold culture, the scaffolds were induced with osteogenic medium for 21 days. (**A**) Alizarin red S staining and (**B**) mineralization quantitation. Compared with the ctrl group, * *p* < 0.05 by one-way ANOVA. n = 3. (Scale bar: A, 100 µm).

Alizarin red S staining for calcium salt nodules usually reflects mineralization and osteogenic activity [31]. After 21 days of osteogenic induction culture, compared with the control group, the alizarin red S staining was enhanced in the Wnt3a group and decreased in the ICG-001 group (Figure 5A). Quantitative analysis showed that calcium deposition in the Wnt3a group was 3.26 times higher than that in the control group (*p* < 0.05) and 8.63 times of the ICG-001 group (*p* < 0.05) (Figure 5B). These results suggest that activation of Wnt signaling promoted ST2 cells’ mineralization, whereas inhibition of Wnt signaling decreased ST2 cells’ mineralization.

### 2.5. Wnt3a Pretreated Bone Repair Scaffold Accelerates Critical-Size Bone Defect Repair

**Figure 6:**
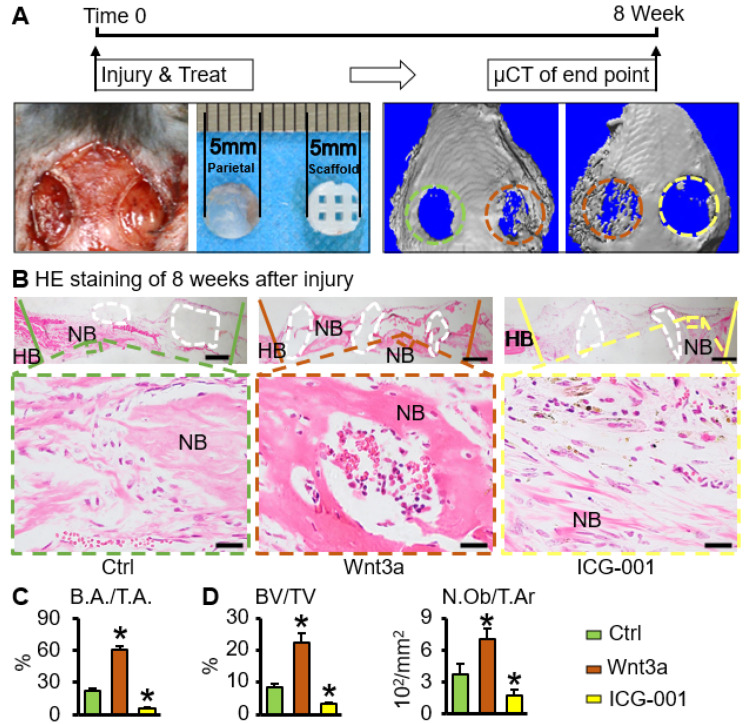
Wnt3a pretreated ST2/GelMA/PCL scaffold accelerates bone regeneration in mice critical-size bone defects. (**A**) Animal experiment. Critical-size bone defect (5 mm in diameter) was established in the parietal of the 6-week-old male mice, and then the scaffolds were implanted. μCT image of bone defect at 8 weeks after surgery (green circle is the ctrl group, the brown circle is the Wnt3a group, the yellow circle is the ICG-001 group) and (**C**) quantitative analysis of the bone area per the tissue area (B.A./T.A.). (**B**) HE staining of the tissue section at the bone defect; the white dotted area is polycaprolactone. HB, host bone. NB, New bone. (**D**) Static histomorphometric analysis, bone volume per tissue volume (BV/TV), osteoblast number per tissue area (N.Ob/T.Ar). Small figure, scale bar: 400 µm. Magnification image, scale bar: 20 µm. Compared with ctrl group, * *p* < 0.05 by one-way ANOVA, n = 5.

The critical-size bone defect of mice parietal bone was used to verify the repair effect of bone tissue engineering scaffolds in vivo [32]. Eight weeks after surgery, compared with the control group, μCT results showed that the Wnt3a group had the best bone repair effect, and the ICG-001 group had the weakest effect (Figure 6A,C). No ectopic bone formation was seen on the HE staining of the tissue section at the bone defect position (Figure 6B). Compared with the control group, the bone tissue volume fraction (BV/TV) in the Wnt3a group (Wnt3a group vs. ctrl group: (22.43 ± 3.07)% vs. (8.48 ± 0.91)%, *p* < 0.05), and osteoblast number per tissue area (N.Ob/T.Ar) in the Wnt3a group (Wnt3a group vs. ctrl group: (7.13 ± 0.93) 10^−2^/mm^2^ vs. (3.78 ± 0.95) 10^−2^/mm^2^, *p* < 0.05) increased significantly. However, BV/TV (3.35 ± 0.52)% and N.Ob/T.Ar (1.73 ± 0.59) 10^−2^/mm^2^ in the ICG-001 group were significantly lower than those in the control group (*p* < 0.05) (Figure 6D). These suggest that the functionalized ST2/GelMA/PCL bone repair scaffold promoted the repair of parietal critical-size bone defects in mice.

### 2.6. Wnt3a Pretreated Bone Repair Scaffolds Promote Vascularization

**Figure 7:**
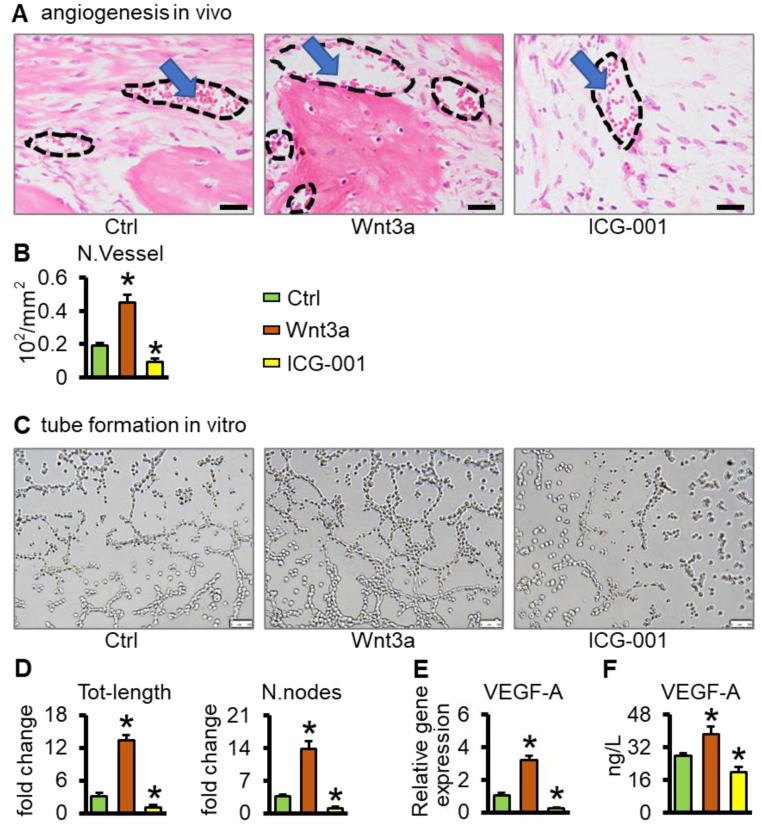
Wnt3a pretreated ST2/GelMA/PCL scaffolds promote vessel formation. (**A**) H&E staining to detect vessel morphology (black dashed area), and blue arrows (red blood cells). (**B**) Quantitative analysis of the number of vessels per unit area (N. Vessel/mm^2^) (n = 5). In vitro tubule formation experiments, the culture supernatant of the scaffolds was co-cultured with human umbilical vein endothelial cells (HUVECs) on the matrix for 6 h. (**C**) Optical images and (**D**) quantitative analysis of the total length of tubes and the number of tubes in each field (n = 3). The amount of VEGF-A in the cells (**E**) and conditioned medium (**F**) of the ST2/GelMA/PCL scaffolds, (n = 3). Compared with ctrl group, * *p* < 0.05 by one-way ANOVA. (Scale bars: A, 20 µm, C, 100 µm).

Eight weeks after surgery, there was a large amount of neovascularization in the Wnt3a group compared with the control group in bone tissue sections (Figure 7A). Quantitative analysis of blood vessels showed that the Wnt3a group was 2.39 times (*p* < 0.05) higher than that of the control group and 4.78 times higher than that of the ICG-001 group (*p* < 0.05) (Figure 7B). Furthermore, Wnt3a increased the amount of VEGF-A in cells (Figure 7E) and conditioned medium (Figure 7F) of the ST2/GelMA/PCL scaffolds, which promoted tubule formation of HUVEC cells in vitro (Figure 7C,D). It is shown that the functionalized ST2/GelMA/PCL bone repair scaffold promotes vascularized functional bone formation.

## 3. Discussion

For standard bone tissue engineering, three elements are required, i.e., a scaffold, growth factors, and seed cells. BMSCs are the most used seed cells, and the regeneration strategy should effectively promote their osteogenic differentiation and angiogenesis. In this study, ST2/GelMA/PCL scaffolds were fabricated by 3D printing and pretreated with Wnt3a (50 ng/mL) for 24 h in vitro. Based on the data of cell proliferation, osteogenic gene expression, mineralization, μCT, and histology analysis, we demonstrated that Wnt3a pretreatment increased the proliferation, osteogenic differentiation, and mineralization of ST2 cells in the scaffolds in vitro, and promoted bone regeneration and angiogenesis in vivo. Simply implanting materials or inoculating cells on the surface of materials before transplantation, it is difficult to make cells grow into materials. Unlike that, 3D bioprinting generates customized macroporous implants for biomaterials and cells to simulate a 3D growth environment and make cells orientated and evenly distributed in scaffolds [33]. In our scaffolds, PCL and GelMA have high biocompatibility, and PCL has been approved for clinical use by FDA [34,35]. ST2 cells survival and proliferation were evenly distributed in the structure, with a high survival rate (above 92%) and proliferation rate.

When analyzed for their osteoinductive potential in vitro, ST2/GelMA/PCL bone repair scaffolds were pretreated with the growth factor Wnt3a in vitro, as previously reported in the Wnt-mediated BMSCs stimulation model [25,36]. We hypothesized that ST2/GelMA/PCL scaffolds pretreated with Wnt3a in vitro were sufficient to trigger osteogenic responses. First, the canonical Wnt/β-catenin signaling pathway is activated in bone marrow stromal cells leading to bone regeneration after trauma [37]. Second, Wnt3a pretreatment of ST2 cells for 24 h was sufficient to enhance the expression of osteoblast transcription factors (Runx2 and Osx) and the formation of mineralization. Similarly, Aquino-Martinez et al. also found that Wnt3a could enhance the expression of Runx2 and Osx in bone marrow stem cells [36]. Finally, ICG-001 reduced the expression of osteogenic differentiation transcription factors in ST2 cells. Zhou et al. reported that the inhibition of Wnt/β-catenin by ICG-001 significantly decreased osteogenic differentiation of bone marrow stromal cells [38]. These results suggest that Wnt/β-catenin is an essential factor in maintaining the osteogenic differentiation of ST2 cells.

Here, we have employed the well-defined mice parietal critical-size bone defect model to evaluate Wnt3a-pretreated ST2/GelMA/PCL scaffolds functionality in vivo. Histological analysis showed significant increases in bone volume per tissue volume (BV/TV), and osteoblast number per tissue area (N.Ob/T.Ar) in the Wnt3a group than in the control group. In addition, according to literature reports, the concentration of Wnt3a in serum is about 1–2 ng/mL [39,40], while the Wnt3a concentration of 25–100 ng/mL is often used in the experiments of inducing osteogenic differentiation in vitro [41,42,43]. However, 40,000 ng/mL of Wnt3a was required for promoting the repair of parietal bone defects in vivo [44]. Different from the direct use of Wnt3a protein for bone repair, ST2/GelMA/PCL scaffolds were pretreated with 50 ng/mL Wnt3a for 24 h in vitro and washed with phosphate-buffered saline before transplantation in this study. This means facilitating the bone formation of critical-size bone defects and avoiding supraphysiological concentration of growth factors used in vivo.

Vascularization is directly related to the final success of newly regenerated bone [45]. McBride et al. reported that bone marrow stromal cells promote angiogenesis through Wnt3a [46]. Activation of Wnt/β-catenin signaling in mesenchymal stem cells is critical for angiogenesis, while the Wntβ-catenin inhibitor ICG-001 can reverse this process [47]. VEGF effectively promotes the coupling of angiogenesis and bone formation in bone repair. Activation of the Wnt signaling pathway leads to up-regulation of VEGF expression, which induces neovascularization, while inhibition of the VEGF signaling pathway inhibits angiogenesis [48]. In this study, VEGF-A expression was up-regulated in Wnt3a pretreated ST2 cell-loaded scaffolds, whose conditioned medium promoted vascular endothelial tubule formation in vitro, and scaffold implantation in bone defects promoted vascularized functional bone formation in vivo, while Wnt/β-catenin signaling inhibitor ICG-001 reduced tubule formation in vitro and angiogenesis in vivo. Dual cell implantation—for example, mesenchymal stromal cells and endothelial progenitor cells—promote bone formation and angiogenesis synergistically [49]. However, as the number of components in the scaffold increases, so does the complexity of the procedure and uncertainty about the treatment outcome [24]. The method reported in this study showed that the single-cell type (ST2) scaffolds induced by Wnt3a in vitro promoted bone formation and angiogenesis, making clinical transformation easier.

## 4. Materials and Methods

### 4.1. Cell Culture

To determine the appropriate concentration of Wnt3a-induced osteogenic differentiation of ST2 cells, ST2 cells (ATCC, Manassas, VA, USA) were expanded in the basal medium (α-MEM medium containing 10% fetal bovine serum (BI, Herzliya, Israel), 100 u/mL penicillin G, and 100 mg/mL streptomycin (Gibco, Gaithersburg, MD, USA)). ST2 cells were planted with 1 × 10^4^/well in 24-well plates and cultured at 37 °C with 5% CO_2_ for 4 h until the cells adhered to the wall. Then, the cells were incubated with different concentration of Wnt3a (0, 25, 50, 100 ng/mL) (R & D Systems, Minneapolis, MN, USA), or ICG-001 (0, 5, 10, 20 μM) (MCE, Dallas, TX, USA) for 24 h. After being rinsed with phosphate-buffered saline (PBS) 3 times, the cells were cultured with the basal medium, and half of the medium was changed every 2 days. On the seventh day, total RNA was extracted and the expression of osteogenic marker Alp and angiogenic factor VEGF-A was detected by qRT-PCR. Methods and primer sequences are described later. The osteogenic differentiation and expression of VEGF-A in ST2 cells were significantly promoted by Wnt3a at 50 ng/mL, while significantly inhibited by ICG-001 at 20 μM. Thus, these concentrations were selected for subsequent experiments.

### 4.2. 3D-Bioprinting and Culture of ST2/GelMA/PCL Scaffolds

GelMA (Sunpbiotech, Beijing, China) was fully dissolved in α-MEM medium to create a solution at the concentration of 20% (*w*/*v*). Then, ST2 cells were trypsinized and resuspended in the basal medium. After mixing them, lithium phenyl-2, 4, 6-trimethylbenzoylphosphinate (LAP) (Sunpbiotech, Beijing, China) was added. The final concentration of ST2 cells, GelMA, LAP was 10^6^/mL, 10% (*w*/*v*) and 0.25% (*w*/*v*), respectively. PCL (Sigma, Hesse, Germany) was sterilized by UV irradiation for 1 h in advance. Hard biomaterials and a cell-integrated 3D system were designed by the Laboratory of Bone Development and Regeneration, Chongqing Medical University, referring to previously reported work [21]. The spiral nozzle loaded with PCL material and the bio-cartridge loaded with ST2 were controlled by the computer to move in the X, Y, and Z axis directions, respectively. Before printing, PCL was put in a molten screw extrusion nozzle and melted at 95 °C for 10 min. At the same time, the ST2-loaded GelMA solution was placed into a syringe and pre-cooled at 4 °C for 5 min, and then the syringe was put in the pneumatic extrusion nozzle for the following printing. Then, the melted PCL was plotted at 300 µm diameter at a printing speed of 3 mm/s. As shown in Figure 2A, the PCL was printed as the frame structure of the scaffold, and then the ST2-loaded GelMA solution was plotted between the PCL strips with a 400 µm interval between cell bundles at a printing speed of 5 mm/s. Each printed layer was irradiated with blue light at 405 nm for 5 s to stabilize and solidify. After printing, the scaffolds were soaked in sterile PBS for 1 min and repeated 3 times to remove excess LAP photoinitiator. The scaffolds were cultured in the basal medium of 10% fetal bovine serum, 100 u/mL penicillin G, and 100 mg/mL streptomycin at 37 °C, 5% CO_2_ for 4 h, and then incubated with Wnt3a (50 ng/mL, final concentration) or ICG-001 (20 μM, final concentration) for 24 h. Next, the scaffolds were rinsed 3 times with PBS before implantation in vivo or subsequent culture in vitro. Half of the medium was changed every two days.

### 4.3. Cell Viability

Cell viability in Wnt3a pretreated scaffolds was determined on days 1, 4, and 7 using the LIVE/DEAD kit (ThermoFisher Scientific, Waltham, MA, USA) as previously described [50]. Briefly, the supernatant was discarded by aspiration, and the scaffolds were washed twice with PBS. Each scaffold was completely soaked in 300 μL LIVE/DEAD staining solution and incubated at 37 °C with 5% CO_2_ for 1 h. Images were taken with a fluorescence microscope (Leica, DMI8, Wetzlar, Germany). Live cells were stained green, while dead cells were stained red. The percentage of viable cells to total cells was calculated with ImageJ software v1.8.0.

### 4.4. Cell Proliferation

The proliferation of cells in the scaffolds was detected on days 1, 4, and 7 with a CCK-8 kit (MCE, Dallas, TX, USA) as previously described [51]. Before detection, ST2 cells were labeled with 5 μL/mL Dio dye and incubated at 37 °C with 5% CO_2_ for 2 h. Next, they were washed 3 times with PBS to remove excess dye. After taking photos quickly by fluorescence microscope, 1 mL of CCK-8 dilution solution was added to each scaffold and incubated at 37 °C with 5% CO_2_ for 2 h. An enzyme plate analyzer was used to detect the absorbance of the incubation solution at 450 nm.

### 4.5. Cell Osteogenic Differentiation Assays

On the seventh day of culture, the scaffolds were stained by BCIP/NBT alkaline phosphatase chromogenic kit (Beyotime Biotechnology, Shanghai, China) as previously described [52]. Cell lysates were collected, and alkaline phosphatase activity was detected with an alkaline phosphatase assay kit (Beyotime Biotechnology, Shanghai, China). The ultrasonic breaker was used to break the cells, centrifuge (12,000× *g*, 5 min), and collect the supernatant. The BCA protein method was used to measure the absorbance at a wavelength of 562 nm, and the protein content of the sample was calculated according to the standard curve. At the same time, an alkaline phosphatase kit was used to measure the absorbance at a wavelength of 405 nm, and the action time was recorded. The amount of AP substance was calculated based on the standard substrate concentration curve. Finally, the relative activity of AP was calculated through the concentration of protein and AP, and the duration of action.

Total RNA was isolated by TRIzol (Invitrogen, Waltham, MA, USA). cDNA synthesis was performed using the corresponding kits (Takara, Tokyo, Japan). The expression of osteogenic differentiation genes was evaluated by qRT-PCR. mRNA expression level was normalized by GAPDH, and expression was calculated by the 2^−ΔΔCt^ method. The forward and reverse primer (Sangon Biotech, Shanghai, China) sequences (5′→3′) were as follows: Alp, CACGGCGTCCATGAGCAGAAC and CAGGCACAGTGGTCAAGGTTGG; Runx2, AACAGCAGCAGCAGCAGCAG and GCACOAGCACAGGAAGTTGG; Col1a1, GAGCGGAGAGTACTGGATCG and GCTTCTTTTCCTTGGGGTTC; Osx, TCGTCTGACTGCCTGCCTAGTG and CTGCGTGGATGCCTGCCTTG; Ocn, AGACTCCGGCGCTACCTTGG and CGGTCTTCAAGCCATACTGGTCTG; VEGF-A, CGAAGCTACTGCCGTCCGATTG and ACGGCTACTACGGAGCGAGAAG; β-catenin, CAAACTGCGTGGATGGGATCTG and AGGGTCCGAGCTGCCATGTT; GAPDH, GCACAGTCAAGGCCGAGAAT and GCCTTCTCCATGGTGGTGGTGAA.

### 4.6. Mineralization Assay

Matrix mineralization in ST2/GelMA/PCL scaffolds was analyzed by alizarin red S (ARS) (Sigma, Burlington, MA, USA) staining, referring to the methods reported in previous studies [53]. After the seventh day of culture, the scaffolds were incubated with osteogenic induction medium (basal medium containing 50 μg/mL ascorbic acid, 5 mM sodium β-glycerophosphate, 1 nM dexamethasone) for 21 days, and half of the medium was changed every two days. Scaffolds were then stained with 1% alizarin red S staining solution. Mineralization was quantified according to the reported method [54]. Briefly, the solution was collected after the scaffolds were incubated with 10% acetic acid for 12 h, and then neutralized with 10% ammonium hydroxide. The absorbance of the solution was measured at a wavelength of 405 nm.

### 4.7. Animal Experiments

The mice parietal critical-size bone defect model was used to evaluate the bone repair effect of the scaffold in vivo [55]. Six-week-old male C57BL/6 mice were used for animal experiments. Mice were fed with a regular diet, received water ad libitum, and were maintained on a 12-h light/dark cycle. Mice were randomly divided into 2 groups with 5 mice in each group: (1) ctrl (lift) and Wnt3a (right); (2) Wnt3a (lift) and ICG-001 (right). Briefly, mice were anesthetized with isoflurane. A trephine (MDJ090729, TUUME, China) was used to create a 5 mm diameter critical-size bone defect on both sides of the skull under low-speed drilling. A large saline flush was used to lower the temperature, and the surgery must be performed carefully to avoid damage to the dura and brain. The scaffolds were then implanted in the defect and the incision was sutured. After the operation, the mice were subcutaneously injected with tramadol (0.1 mg·g^−1^) to relieve pain. All animal experiments were approved by the Animal Care and Use Committee of Chongqing Medical University, and in accordance with institutional animal guidelines.

### 4.8. Analysis of Bone Regeneration In Vivo

Specimens from each group were taken 8 weeks after implantation. Mice in each group were sacrificed by cervical dislocation after inhaling CO_2_. The entire skull including the implants was surgically removed and fixed with 4% paraformaldehyde at 4 °C for 24 h. The parietal bone was scanned with nanoScan CT (Mediso, Budapest, Hungary) at 50 kV energy and 165 ms intensity. The PMOD (Zurich, Switzerland) software (version 4.3, threshold value, 500) [56] was used for the reconstruction of regenerated bone tissue. The area of interest was defined as a dotted circle with a diameter of 5 mm. The new bone formation was evaluated by the ratio of bone area and tissue area (B.A./T.A.). Then, specimens were decalcified in 10% EDTA (pH 7.4) for 21 days. After dehydration, the specimens were embedded in paraffin and cut into 5 μm thick tissue sections, which were stained with hematoxylin and eosin (H&E) to observe the healing and internal defects of different groups. The number of osteoblasts in the new bone and the area of the blood vessels containing red blood cells (RBCs) was counted by OsteoMeasure™ software (v4.1.0.2, OsteoMetrics, Knoxville, TN, USA). The terms and units used are those recommended by the Organizational Morphometric Nomenclature Committee of the American Society for Bone and Mineral Research [57].

### 4.9. Tubular Formation Experiment

According to a previously reported method [58], ST2/GelMA/PCL scaffold-induced tubule formation in HUVECs was performed in vitro. HUVECs (2 × 10^5^ cells/cm^2^) were mixed with 4-fold diluted cell supernatants and seeded on matrix (200 μL)-coated 24-well plates for 6 h. Capillary-like structures were counted with a microscope. The tube-forming network of HUVECs was quantified with Image-J.

### 4.10. ELISA

Angiogenic factor (VEGF-A) in scaffold culture supernatants was detected by ELISA. Sample supernatants were centrifuged at 1000× *g* for 5 min to remove cellular debris, and VEGF-A in scaffold culture supernatants was accurately measured using a mouse ELISA kit according to the manufacturer’s instructions. The absorbance at 450 nm was measured by a Bio-Rad Microplate Analyzer (Hercules, CA, USA). A standard curve for the assay was established using cytokine standards provided by ELISA kits (Beyotime Biotechnology, Shanghai, China).

### 4.11. Statistical Analysis

Data were analyzed using GraphPad software (Chicago, IL, USA). One-way ANOVA was followed by Tukey’s post hoc test. The final results are presented as the mean ± standard deviation of 3–5 experiments per group. For all tests, *p*-values less than 0.05 were considered statistically significant.

## 5. Conclusions

In conclusion, the 3D-printed ST2/GelMA/PCL bone repair scaffold pretreated with Wnt3a for 24 h in vitro was used to fabricate a single-cell scaffold with dual functions of osteogenesis and angiogenesis in this study, which could realize the mass and stable production of functional materials for bone regeneration and improve bone regeneration and angiogenesis in animal models with bone defects. It avoids the use of supraphysiological concentrations of growth factors in vivo and the resulting side effects, thus providing an outstanding bone substitute for bone regeneration and repair.

## Data Availability

All data generated or analyzed during this study are included in this published article.

## References

[1] Turnbull G., Clarke J., Picard F., Riches P., Jia L., Han F., Li B., Shu W. (2018). 3D bioactive composite scaffolds for bone tissue engineering. Bioact. Mater..

[2] Perez J.R., Kouroupis D., Li D.J., Best T.M., Kaplan L., Correa D. (2018). Tissue Engineering and Cell-Based Therapies for Fractures and Bone Defects. Front. Bioeng. Biotechnol..

[3] Bjelić D., Finšgar M. (2021). The Role of Growth Factors in Bioactive Coatings. Pharmaceutics.

[4] Wang L., Bai J., Wang Q., Ge G., Lin J., Xu N., Xu C., Xu Y., Wang Y., Geng D. (2020). Inhibition of protein phosphatase 2A attenuates titanium-particle induced suppression of bone formation. Int. J. Biol. Macromol..

[5] Nambiar J., Jana S., Nandi S.K. (2022). Strategies for Enhancing Vascularization of Biomaterial-Based Scaffold in Bone Regeneration. Chem. Rec..

[6] Kolan K.C.R., Li J., Roberts S., Semon J.A., Park J., Day D.E., Leu M.C. (2019). Near-field electrospinning of a polymer/bioactive glass composite to fabricate 3D biomimetic structures. Int. J. Bioprint..

[7] Ebrahimi S., Hanim Y.U., Sipaut C.S., Jan N.B.A., Arshad S.E., How S.E. (2021). Fabrication of Hydroxyapatite with Bioglass Nanocomposite for Human Wharton’s-Jelly-Derived Mesenchymal Stem Cell Growing Substrate. Int. J. Mol. Sci..

[8] Wu Y.-H.A., Chiu Y.-C., Lin Y.-H., Ho C.-C., Shie M.-Y., Chen Y.-W. (2019). 3D-Printed Bioactive Calcium Silicate/Poly-ε-Caprolactone Bioscaffolds Modified with Biomimetic Extracellular Matrices for Bone Regeneration. Int. J. Mol. Sci..

[9] Chen Y.-W., Shen Y.-F., Ho C.-C., Yu J., Wu Y.-H.A., Wang K., Shih C.-T., Shie M.-Y. (2018). Osteogenic and angiogenic potentials of the cell-laden hydrogel/mussel-inspired calcium silicate complex hierarchical porous scaffold fabricated by 3D bioprinting. Mater. Sci. Eng. C.

[10] Fitzpatrick V., Martín-Moldes Z., Deck A., Torres-Sanchez R., Valat A., Cairns D., Li C., Kaplan D.L. (2021). Functionalized 3D-printed silk-hydroxyapatite scaffolds for enhanced bone regeneration with innervation and vascularization. Biomaterials.

[11] Wang W., Yeung K.W.K. (2017). Bone grafts and biomaterials substitutes for bone defect repair: A review. Bioact. Mater..

[12] Seims K.B., Hunt N.K., Chow L.W. (2021). Strategies to Control or Mimic Growth Factor Activity for Bone, Cartilage, and Osteochondral Tissue Engineering. Bioconjug. Chem..

[13] Gillman C.E., Jayasuriya A.C. (2021). FDA-approved bone grafts and bone graft substitute devices in bone regeneration. Mater. Sci. Eng. C.

[14] El Bialy I., Jiskoot W., Reza Nejadnik M. (2017). Formulation, Delivery and Stability of Bone Morphogenetic Proteins for Effective Bone Regeneration. Pharm. Res..

[15] Santo V.E., Gomes M.E., Mano J.F., Reis R.L. (2013). Controlled release strategies for bone, cartilage, and osteochondral engineering--Part II: Challenges on the evolution from single to multiple bioactive factor delivery. Tissue Eng. Part B Rev..

[16] Sreekumar V., Aspera-Werz R.H., Tendulkar G., Reumann M.K., Freude T., Breitkopf-Heinlein K., Dooley S., Pscherer S., Ochs B.G., Flesch I. (2017). BMP9 a possible alternative drug for the recently withdrawn BMP7? New perspectives for (re-)implementation by personalized medicine. Arch. Toxicol..

[17] Xiong A., He Y., Gao L., Li G., Weng J., Kang B., Wang D., Zeng H. (2020). Smurf1-targeting miR-19b-3p-modified BMSCs combined PLLA composite scaffold to enhance osteogenic activity and treat critical-sized bone defects. Biomater. Sci..

[18] Tu X., Delgado-Calle J., Condon K.W., Maycas M., Zhang H., Carlesso N., Taketo M.M., Burr D.B., Plotkin L.I., Bellido T. (2015). Osteocytes mediate the anabolic actions of canonical Wnt/beta-catenin signaling in bone. Proc. Natl. Acad. Sci. USA.

[19] Leucht P., Minear S., Berge D.T., Nusse R., Helms J. (2008). Translating insights from development into regenerative medicine: The function of Wnts in bone biology. Semin. Cell Dev. Biol..

[20] Zhou Y., Snead M.L., Tamerler C. (2015). Bio-inspired hard-to-soft interface for implant integration to bone. Nanomed. Nanotechnol. Biol. Med..

[21] Kang H.-W., Lee S.J., Ko I.K., Kengla C., Yoo J.J., Atala A. (2016). A 3D bioprinting system to produce human-scale tissue constructs with structural integrity. Nat. Biotechnol..

[22] Diomede F., Marconi G.D., Fonticoli L., Pizzicanella J., Merciaro I., Bramanti P., Mazzon E., Trubiani O. (2020). Functional Relationship between Osteogenesis and Angiogenesis in Tissue Regeneration. Int. J. Mol. Sci..

[23] Huang X., Li C., Zhu B., Wang H., Luo X., Wei L. (2016). Co-cultured hBMSCs and HUVECs on human bio-derived bone scaffolds provide support for the long-term ex vivo culture of HSC/HPCs. J. Biomed. Mater. Res. Part A.

[24] Rather H.A., Jhala D., Vasita R. (2019). Dual functional approaches for osteogenesis coupled angiogenesis in bone tissue engineering. Mater. Sci. Eng. C.

[25] Jing W., Smith A.A., Liu B., Li J., Hunter D.J., Dhamdhere G., Salmon B., Jiang J., Cheng D., Johnson C.A. (2015). Reengineering autologous bone grafts with the stem cell activator WNT3A. Biomaterials.

[26] Fuster-Matanzo A., Manferrari G., Marchetti B., Pluchino S. (2018). Wnt3a promotes pro-angiogenic features in macrophages in vitro: Implications for stroke pathology. Exp. Biol. Med..

[27] Zhang J.Y., Lee J.H., Gu X., Wei Z.Z., Harris M.J., Yu S.P., Wei L. (2018). Intranasally Delivered Wnt3a Improves Functional Recovery after Traumatic Brain Injury by Modulating Autophagic, Apoptotic, and Regenerative Pathways in the Mouse Brain. J. Neurotrauma.

[28] Li X., Liu D., Li J., Yang S., Xu J., Yokota H., Zhang P. (2019). Wnt3a involved in the mechanical loading on improvement of bone remodeling and angiogenesis in a postmenopausal osteoporosis mouse model. FASEB J..

[29] Tschaffon M.E.A., Reber S.O., Schoppa A., Nandi S., Cirstea I.C., Aszodi A., Ignatius A., Haffner-Luntzer M. (2022). A novel in vitro assay to study chondrocyte-to-osteoblast transdifferentiation. Endocrine.

[30] Wu Z., Xie S., Kang Y., Shan X., Li Q., Cai Z. (2021). Biocompatibility evaluation of a 3D-bioprinted alginate-GelMA-bacteria nanocellulose (BNC) scaffold laden with oriented-growth RSC96 cells. Mater. Sci. Eng. C Mater. Biol. Appl..

[31] Fang H., Luo C., Liu S., Zhou M., Zeng Y., Hou J., Chen L., Mou S., Sun J., Wang Z. (2020). A biocompatible vascularized graphene oxide (GO)-collagen chamber with osteoinductive and anti-fibrosis effects promotes bone regeneration in vivo. Theranostics.

[32] Li W., Liu Y., Zhang P., Tang Y., Zhou M., Jiang W., Zhang X., Wu G., Zhou Y. (2018). Tissue-Engineered Bone Immobilized with Human Adipose Stem Cells-Derived Exosomes Promotes Bone Regeneration. ACS Appl. Mater. Interfaces.

[33] Amler A.-K., Thomas A., Tüzüner S., Lam T., Geiger M.-A., Kreuder A.-E., Palmer C., Nahles S., Lauster R., Kloke L. (2021). 3D bioprinting of tissue-specific osteoblasts and endothelial cells to model the human jawbone. Sci. Rep..

[34] Ahmadi Lakalayeh G., Rahvar M., Haririan E., Karimi R., Ghanbari H. (2018). Comparative study of different polymeric coatings for the next-generation magnesium-based biodegradable stents. Artif. Cells Nanomed. Biotechnol..

[35] Yang X., Sun X., Liu J., Huang Y., Peng Y., Xu Y., Ren L. (2021). Photo-crosslinked GelMA/collagen membrane loaded with lysozyme as an antibacterial corneal implant. Int. J. Biol. Macromol..

[36] Aquino-Martínez R., Rodríguez-Carballo E., Gámez B., Artigas N., Carvalho-Lobato P., Manzanares-Céspedes M.C., Rosa J.L., Ventura F. (2016). Mesenchymal Stem Cells Within Gelatin/CaSO_4_ Scaffolds Treated Ex Vivo with Low Doses of BMP-2 and Wnt3a Increase Bone Regeneration. Tissue Eng. Part A.

[37] Leucht P., Lee S., Yim N. (2019). Wnt signaling and bone regeneration: Can’t have one without the other. Biomaterials.

[38] Zhou X., Liu Z., Huang B., Yan H., Yang C., Li Q., Jin D. (2019). Orcinol glucoside facilitates the shift of MSC fate to osteoblast and prevents adipogenesis via Wnt/β-catenin signaling pathway. Drug Des. Dev. Ther..

[39] Gong W., Guo M., Han Z., Wang Y., Yang P., Xu C., Wang Q., Du L., Li Q., Zhao H. (2016). Mesenchymal stem cells stimulate intestinal stem cells to repair radiation-induced intestinal injury. Cell Death Dis..

[40] Deng L., Liang H., Han Y. (2020). Cyclooxygenase-2 and β-Catenin as Potential Diagnostic and Prognostic Markers in Endometrial Cancer. Front. Oncol..

[41] Vijaykumar A., Root S., Mina M. (2020). Wnt/β-Catenin Signaling Promotes the Formation of Preodontoblasts In Vitro. J. Dent. Res..

[42] Morsczeck C., Reck A., Reichert T. (2017). WNT3A and the induction of the osteogenic differentiation in adipose tissue derived mesenchymal stem cells. Tissue Cell.

[43] Jiang B., Xu J., Zhou Y., Mao J., Guan G., Xu X., Mei L. (2020). Estrogen Enhances Osteogenic Differentiation of Human Periodontal Ligament Stem Cells by Activating the Wnt/β-Catenin Signaling Pathway. J. Craniofacial Surg..

[44] Moschouris P., Retzepi M., Petrie A., Donos N. (2017). Effect of Wnt3a delivery on early healing events during guided bone regeneration. Clin. Oral Implant. Res..

[45] Liu W.C., Chen S., Zheng L., Qin L. (2017). Angiogenesis Assays for the Evaluation of Angiogenic Properties of Orthopaedic Biomaterials—A General Review. Adv. Healthc. Mater..

[46] McBride J.D., Rodriguez-Menocal L., Guzman W., Candanedo A., Garcia-Contreras M., Badiavas E.V. (2017). Bone Marrow Mesenchymal Stem Cell-Derived CD63(+) Exosomes Transport Wnt3a Exteriorly and Enhance Dermal Fibroblast Proliferation, Migration, and Angiogenesis In Vitro. Stem. Cells Dev..

[47] Zhang B., Wu X., Zhang X., Sun Y., Yan Y., Shi H., Zhu Y., Wu L., Pan Z., Zhu W. (2015). Human umbilical cord mesenchymal stem cell exosomes enhance angiogenesis through the Wnt4/β-catenin pathway. Stem. Cells Transl. Med..

[48] Olsen J.J., Pohl S., Deshmukh A., Visweswaran M., Ward N.C., Arfuso F., Agostino M., Dharmarajan A. (2017). The Role of Wnt Signalling in Angiogenesis. Clin. Biochem. Rev..

[49] Bouland C., Philippart P., Dequanter D., Corrillon F., Loeb I., Bron D., Lagneaux L., Meuleman N. (2021). Cross-Talk Between Mesenchymal Stromal Cells (MSCs) and Endothelial Progenitor Cells (EPCs) in Bone Regeneration. Front. Cell Dev. Biol..

[50] Pan Y., Hu N., Wei X., Gong L., Zhang B., Wan H., Wang P. (2019). 3D cell-based biosensor for cell viability and drug assessment by 3D electric cell/matrigel-substrate impedance sensing. Biosens. Bioelectron..

[51] Wu X., Chen K., Chai Q., Liu S., Feng C., Xu L., Zhang D. (2022). Freestanding vascular scaffolds engineered by direct 3D printing with Gt-Alg-MMT bioinks. Biomater. Adv..

[52] Shao P.-L., Wu S.-C., Lin Z.-Y., Ho M.-L., Chen C.-H., Wang C.-Z. (2019). Alpha-5 Integrin Mediates Simvastatin-Induced Osteogenesis of Bone Marrow Mesenchymal Stem Cells. Int. J. Mol. Sci..

[53] López-González I., Zamora-Ledezma C., Sanchez-Lorencio M.I., Tristante Barrenechea E., Gabaldón-Hernández J.A., Meseguer-Olmo L. (2021). Modifications in Gene Expression in the Process of Osteoblastic Differentiation of Multipotent Bone Marrow-Derived Human Mesenchymal Stem Cells Induced by a Novel Osteoinductive Porous Medical-Grade 3D-Printed Poly(ε-caprolactone)/β-tricalcium Phosphate Composite. Int. J. Mol. Sci..

[54] Yan Y., Chen H., Zhang H., Guo C., Yang K., Chen K., Cheng R., Qian N., Sandler N., Zhang Y.S. (2019). Vascularized 3D printed scaffolds for promoting bone regeneration. Biomaterials.

[55] Reyes R., Rodríguez J.A., Orbe J., Arnau M.R., Évora C., Delgado A. (2018). Combined sustained release of BMP2 and MMP10 accelerates bone formation and mineralization of calvaria critical size defect in mice. Drug Deliv..

[56] Mochizuki M., Güç E., Park A.J., Julier Z., Briquez P.S., Kuhn G.A., Müller R., Swartz M.A., Hubbell J.A., Martino M.M. (2020). Growth factors with enhanced syndecan binding generate tonic signalling and promote tissue healing. Nat. Biomed. Eng..

[57] Delgado-Calle J., Tu X., Pacheco-Costa R., McAndrews K., Edwards R., Pellegrini G.G., Kuhlenschmidt K., Olivos N., Robling A., Peacock M. (2017). Control of Bone Anabolism in Response to Mechanical Loading and PTH by Distinct Mechanisms Downstream of the PTH Receptor. J. Bone Miner. Res. Off. J. Am. Soc. Bone Miner. Res..

[58] Piard C., Jeyaram A., Liu Y., Caccamese J., Jay S.M., Chen Y., Fisher J. (2019). 3D printed HUVECs/MSCs cocultures impact cellular interactions and angiogenesis depending on cell-cell distance. Biomaterials.

